# An integrated RNA sequencing and network pharmacology approach reveals the molecular mechanism of dapagliflozin in the treatment of diabetic nephropathy

**DOI:** 10.3389/fendo.2022.967822

**Published:** 2022-09-21

**Authors:** Zhenyu Bai, Ting Xie, Tianhao Liu, Zedong Chen, Linde Yu, Chao Zhang, Jincheng Luo, Liguo Chen, Xiaoshan Zhao, Ya Xiao

**Affiliations:** ^1^School of Traditional Chinese Medicine, Jinan University, Guangzhou, China; ^2^Department of Gastroenterology, Affiliated Hospital of Jiangnan University, Wuxi School of Medicine, Jiangnan University, Wuxi, China; ^3^GuangDong Province Engineering Technology Research Institute of Traditional Chinese Medicine (TCM), Guangzhou, China; ^4^Emergency Department, GuangDong Second Traditional Chinese Medicine Hospital, Guangzhou, China; ^5^School of Traditional Chinese Medicine, Southern Medical University, Guangzhou, China

**Keywords:** diabetic nephropathy, dapagliflozin, RNA sequencing, network pharmacology, sodium-glucose cotransporter 2

## Abstract

Dapagliflozin, an inhibitor of sodium-glucose cotransporter 2 (SGLT2), is a new type of oral hypoglycemic drugs which can promote glucose excretion in the kidney. Studies have shown that dapagliflozin has renoprotective effect in the treatment of type 2 diabetes. However, the underlying mechanism remains unclear. Here, we combined integrated RNA sequencing and network pharmacology approach to investigate the molecular mechanism of dapagliflozin for diabetic nephropathy (DN). Dapagliflozin significantly relieved glucose intolerance, urinary albumin/creatinine ratio (UACR) and renal pathological injuries of db/db mice. The LncRNA and mRNA expression in kidney tissues from control group (CR), db/db group (DN) and dapagliflozin group (DG) were assessed by RNA sequencing. We identified 7 LncRNAs and 64 mRNAs common differentially expressed in CR vs DN and DN vs DG, which were used to construct co-expression network to reveal significantly correlated expression patterns in DN. In addition, network pharmacology was used to predict the therapeutic targets of dapagliflozin and we constructed component-target-pathway network according to the results of RNA sequencing and network pharmacology. We found that SMAD9, PPARG, CD36, CYP4A12A, CYP4A12B, CASP3, H2-DMB2, MAPK1, MAPK3, C3 and IL-10 might be the pivotal targets of dapagliflozin for treating DN and these genes were mainly enriched in pathways including TGF-β signaling pathway, PPAR signaling pathway, Chemokine signaling pathway, etc. Our results have important implication and provide novel insights into the protective mechanism of dapagliflozin for treating DN.

## Introduction

Diabetes has become a significant public health issue in recent years and is estimated to affect nearly 700 million people worldwide by 2045 (1). Diabetic nephropathy (DN), a major complication of diabetes, can lead to end-stage kidney disease (ESKD) and mainly manifest as hypertrophy, mesangial expansion and thickened basement membrane (2). Persistent hyperglycemia causes renal inflammation, apoptosis and oxidative stress, which may be closely related with DN progression (3). Recently, sodium-glucose transport protein 2 (SGLT2) inhibitors have been developed for the treatment of hyperglycemia (4, 5). Dapagliflozin, a selective inhibitor of SGLT2, can lower blood glucose *via* blocking glucose reabsorption in the renal proximal tubule and stimulating urinary glucose excretion without increasing insulin release (6). Several large clinical trials have been performed to investigate the effect of SGLT2 inhibitors on renal outcomes. In the clinical trial, dapagliflozin significantly reduced renal events (7). Therefore, dapagliflozin can act as a new pharmacologic option for overcoming DN progression in patients with diabetes. Interestingly, dapagliflozin also exerts renoprotective effect and prevents the progression to ESKD regardless of the presence or absence of diabetes (8), indicating that dapagliflozin protect the kidney *via* pleiotropic effects beyond glycemic control. Previous studies have showed that SGLT2 inhibitors can induce tubulo-glomerular feedback, reduce glomerular hyperfiltration and simulate antioxidant and anti-inflammatory signaling pathway (9, 10). However, little is known about the underlying molecular mechanism of dapagliflozin in the treatment of DN.

RNA sequencing (RNA-SEQ) is one of the most advanced techniques to explore the mechanism of various diseases (11, 12). Based on the advantages of high sensitivity and resolution, whole transcriptome sequencing can reveal crucial roles of coding and non-coding RNAs and provide new insights into gene expression changes. Long non-coding RNA (LncRNA) are regulatory RNAs over 200 nucleotides (nt) which does not encode proteins. LncRNA is localized in the nucleus or cytoplasm, and some LncRNA structures are similar to mRNA with polyA tail. Some LncRNAs have conserved secondary structures that can interact with proteins, DNA and RNA and regulate multiple biological processes (13). With the development of RNA-SEQ technology, increasing numbers of LncRNAs have been identified. Studies showed that LncRNA MALAT1 promotes renal fibrosis and injury in DN *in vivo* and vitro (14, 15). Li et al. reported that LncRNATug1/PGC1a has renoprotective effect *via* regulating mitochondrial remodeling and urea cycle metabolites in diabetic mice (16). LncRNA Erbb4-IR was reported to promote renal fibrosis *via* inhibiting miR-29b in DN (17). Although emerging studies showed LncRNAs involved in DN pathogenesis, the functional roles of LncRNAs are largely unknown. Particularly, the regulatory mechanism of LncRNAs and the whole transcriptome in DN treated by dapagliflozin has not been studied.

Network pharmacology has attracted more and more attention. It constructs the connections between components, diseases and signaling pathways and offers a practical approach to elucidate the pharmacological mechanisms of agents (18). It is a powerful tool for establishing a “component-gene-disease” network. Therefore, combining RNA sequencing technologies and network pharmacology is a good strategy to study the mechanisms of dapagliflozin for DN.

Thus, we conducted a comprehensive study to investigated the mechanism of renoprotective effect of dapagliflozin on diabetic mice. First, RNA sequencing was carried out to identify differentially expressed LncRNAs and mRNAs in db/m mice, db/db mice and db/db mice treated with dapagliflozin. Then, co-expression network of differentially expressed LncRNAs/mRNAs was established. Finally, combining with network pharmacology and bioinformatics analysis, component- target-pathway network was constructed to identify the key genes targeted by dapagliflozin.

## Materials and methods

### Animal model and treatments

C57BL/KsJ leptin receptor-deficient (db/db) and db/m male mice were provided by the Model Animal Research Institute of Nanjing University. The animals were 4-6 weeks old. Six db/m mice comprised control group (CR). Twelve db/db mice were randomly divided into two groups: the db/db group (DN, n=6) and db/db+ Dapagliflozin group (DG, n=6). Mice in DG group were fed 50mg/Kg dapagliflozin-supplemented diet, while mice in CR and DN group were fed control diet. Dapagliflozin was from AstraZeneca Pharmaceuticals LP. After twelve-week dietary intervention, the mice were anaesthetized with sodium pentobarbital (60 mg/kg iP). Body weight was recorded once a week and oral glucose tolerance test was conducted once four weeks. Blood was collected from orbital venous and kidney tissues were collected for further study. Blood glucose was measured by blood glucose metre (Roche Diabetes Care GmbH, UK). The experiment was approved by the Animal Experiment Ethics Committee of Jinan University.

### Serum and urine biochemistry assays

Serum creatinine levels and blood urea nitrogen (BUN) were assessed using the Quanti Chrom Creatinine Assay Kit (BioAssay Systems, USA). Urinary albumin was measured using the mouse urinary albumin ELISA kit (Bethyl Laboratories, USA). Urinary albumin/creatinine ratio (UACR) was calculated as urinary albumin/creatinine ratio.

### Oral glucose tolerance test

The test was measured at fourth week, eighth week and twelfth week. First, the mice were fasted for 6 hours and received glucose solution with 2.0 g/kg by gavage. Finally, the blood glucose level was tested at 0, 30, 60, 90, and 120 minutes.

### Histopathology

The kidneys were harvested and fixed in 4% paraformaldehyde for 1-2h. Then, the samples were dehydrated, immersed in xylene and embedded in paraffin. The samples were cut into 5 μm thick sections and used for hematoxylin-eosin (HE), Periodic acid-Schiff (PAS) and Masson staining. All sections were observed by a microscope (Olympus, Tokyo, Japan) and assessed by Image-Pro Plus 6.0 software (Media Cybernetics, Bethesda, MD).

### Total RNA extraction, library construction and sequencing

Total RNA was extracted from renal tissues in three groups (n = 3 for each) by TRIzol (Invitrogen) reagent. RNA purity and RNA integrity were assessed by Agilent 2200 TapeStation (Agilent Technologies, USA). Ribosomal RNA was eliminated using Ribo-Zero™ kit (Epicentre, Madison, WI, USA) and fragmented to approximately 200bp. Subsequently, the purified RNAs were used to synthesis first strand and second strand cDNA according to instructions of NEBNext^®^ Ultra™ RNA Library Prep Kit for Illumina (NEB, USA). The purified library products were assessed by Agilent 2200 TapeStation and Qubit^®^2.0(Life Technologies, USA). The libraries were paired-end sequenced at Guangzhou RiboBio Co., Ltd. (Guangzhou, China) using IlluminaHiSeq 3000 platform.

### Identification of new LncRNA

After removing low-quality reads, the clean data was assembled using the StringTie based on the reads mapped to the reference genome. Gffcompare program was used to annotate the transcripts. Putative protein-coding RNAs were filtered out using a minimum length and exon number threshold. Transcripts with lengths between 200 nt and 300 nt could be selected as LncRNA candidates, which were submitted to further screening by CPC/CNCI/Pfam to distinguish the protein-coding genes from the noncoding genes.

### Differentially expressed mRNA and LncRNA Genes (DEGs and DELncRNAs)

Paired-end reads were aligned to the mouse reference genome mm10 with HISAT2. HTSeq v0. 6.0 was used to count the reads numbers mapped to each gene. RPKM was used to evaluate the sample expression level (expected number of Reads PerKilobase of transcript sequence per Million base pairs sequenced). An adjusted P-value threshold of <0.05 and |log2(fold change)| > 1 was utilized to identify differentially expressed genes.

### Co-expression network of differentially expressed LncRNAs/mRNAs

To investigate the relationships between LncRNAs and mRNAs, we constructed a LncRNA/mRNA transcripts co-expression network. A given threshold (absolute Pearson correlation coefficients no less than 0.7 and p-values less than 0.05) was used to filter the results. The co-expression network was illustrated by Cytoscape software.

### GO terms and KEGG pathway enrichment analysis

Differentially expressed mRNAs were utilized for Gene ontology (GO) functional enrichment and Kyoto Encyclopedia of Genes and Genomes (KEGG) pathway analyses by the KOBAS3.0 software. A P-value < 0.05 was determined to be significant in the enrichment analysis of the gene sets.

### Network pharmacological analysis of therapeutic pathways and targets of dapagliflozin in the treatment of DN

Firstly, the bioactive component and potential pharmacological targets of dapagliflozin were obtained by CHEMBL, STITCH, Drugbank, Pubchem and PubMed. Secondly, we got the pathogenic targets of DN through searching for the keyword “diabetic nephropathy” in GeneCards, Online Mendelian Inheritance in Man and PharmGkb databases. Thirdly, we intersected the above two sets to obtain potential targets of dapagliflozin in DN by the Venny2.1 online tool. Fourthly, a protein-protein interaction (PPI) network was constructed by STRING online database based on the obtained target genes. The top 20 target genes were selected to perform KEGG and GO enrichment analysis.

### Construction of component- target-pathway network

A component- target- pathway network was constructed to obtain the key targets and pathway of dapagliflozin in DN by Cytoscape3.8.2 based on the data of KEGG enrichment analysis of RNA sequencing and network pharmacology.

### Quantitative real-time PCR

Total RNA was extracted from the kidneys using TRIzol kit (Invitrogen, Carlsbad, CA). An Invitrogen (Carlsbad, CA) kit was used to perform reverse transcription. Real-time quantitative RT-PCR was conducted by the Bio–Rad 96FX circulation system (Bio-Rad, USA) with SYBR Green Master Mix. The relative expression levels of genes were calculated by the 2-ΔΔCq method. The primers for genes for qRT-PCR were listed in **Table S1**.

### Western blotting

Proteins were extracted from renal tissues using RIPA method. A BCA protein detection kit was used to measure protein concentrations. After subjecting to SDS-PAGE, proteins were transferred to nitrocellulose membranes. Then, membranes were incubated with primary antibodies overnight as follows: anti-IL-10 (1:1000,ab189392), anti-PPAR gamma (1:1000, ab272718), anti-CD36 (1:1000, ab252923), anti-Caspase-3 (1:2000, ab184787), anti-MAPK1 (1:2000, ab32081), anti-MAPK3 (1:1000, ab32537), anti-C3 (1:3000, ab97462), anti-SMAD9 (1:1000, ab80255), anti-GAPDH (1:500, ab8245). Membranes were incubated with the HRP-conjugated secondary antibodies. Gel-pro analyser software was used to analysis the images.

### Statistical analysis

GraphPad Prism 7 software was used for statistical analysis. The data were expressed as the mean ± SE. Comparisons of multiple groups were performed by one-way ANOVA. Student’s t-test was used to determine significant differences between two independent groups. P<0.05 was considered statistically significant.

## Results

### Dapagliflozin ameliorated renal injury in DN mouse model

**Figure 1:**
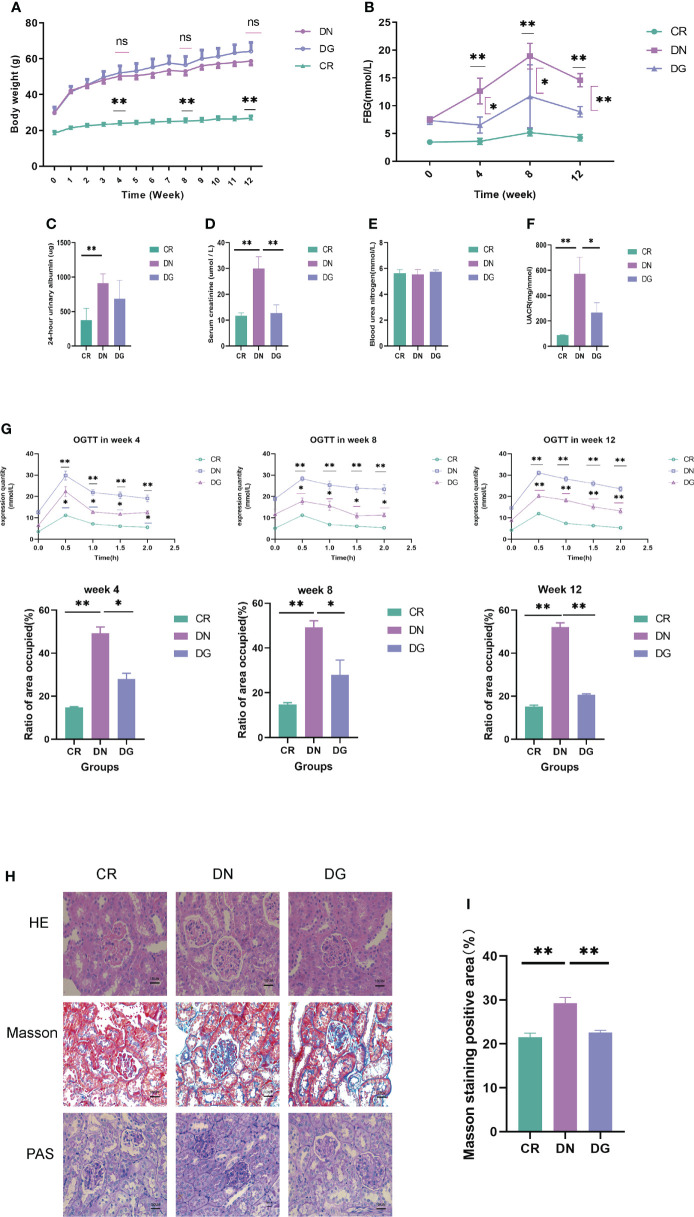
Dapagliflozin improved kidney injury in db/db mice. **(A) **The body weights recorded every week (n=6). **(B)** Quantitative analysis of fasting blood glucose (FBG) (n=6). **(C)** Quantitative analysis of 24-hour urinary albuamin (n=4-5). **(D)** Quantitative analysis of serum creatinine (SCR) (n=5). **(E)** Quantitative analysis of blood urea nitrogen (BUN) (n=5). **(F)** Quantitative analysis of urinary albumin/creatinine ratio (UACR) (n=4). **(G)** The levels of OGTT and AUC at week4, week8 and week12 (n=6). **(H-I)** The pathologic changes in kidneys *via* HE, PAS and Masson staining. Data are presented as the mean ± SE. *P < 0.05; **P < 0.001. AUC, Area under the curve; CR, control group; DN, diabetic nephropathy group; DG, dapagliflozin group; OGTT, Oral glucose tolerance test. ns, no significant.

Body weight, 24-hour urinary protein, UACR, BUN, serum creatinine, fasting blood glucose (FBG) and glucose tolerance were used to assess the effect of dapagliflozin in DN mouse model. Mice in DN group and dapagliflozin group had markedly increased body weight compared with that in CR group **(** **Figure 1A****)**. Db/db mice had significantly high FBG, 24-hour urinary albumin, BUN, serum creatinine, UACR and obvious glucose intolerance. The dapagliflozin group showed markedly reduced FBG, serum creatinine, UACR and improved glucose intolerance comparing to db/db mice models **(** **Figures 1B–G****)**. Dapagliflozin tended to decrease 24-hour urinary albumin, but this effect failed to reach statistical significance.

As to pathological changes, HE staining showed that db/db mice had glomerular enlargement and glomerular capillary loops dilatation. PAS and Masson staining revealed that db/db mice had mesangial matrix, mesangial expansion, thickened basement membrane and increased renal fibrosis. Treatment with dapagliflozin alleviated these pathological injuries **(** **Figures 1H****)**. Quantitative analysis also showed statistically significant differences in pathology **(** **Figures 1I****)**. Taken together, dapagliflozin can improve glucose intolerance, UACR and kidney injuries.

### Identification of differentially expressed LncRNAs in DN

**Figure 2:**
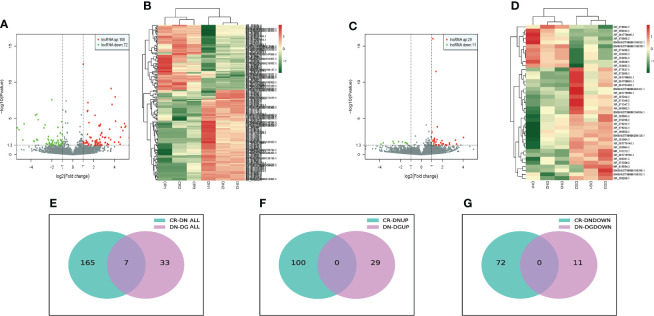
Differentially expressed LncRNAs in DN (n=3). **(A, B)** Volcano plots **(A)** and heatmaps **(B)** for comparison between CR and DN. **(C, D)** Volcano plots**(C)** and heatmaps **(D)** for comparison between DN and DG. **(E)** Overlap of the differentially expressed LncRNAs in the comparisons between CR vs DN and DN vs DG. **(F)** Upregulated differentially expressed LncRNAs in the comparisons between CR vs DN and DN vs DG. **(G)** Downregulated differentially expressed LncRNAs in the comparisons between CR vs DN and DN vs DG. CR, control group; DN, diabetic nephropathy group; DG, dapagliflozin group.

The bioinformatics analysis was divided into two independent comparisons:CR vs DN and DN vs DG. Volcano plots and heat map provided an overview of LncRNAs differential expressed in the two comparisons **(** **Figures 2A–D****)**. Venn diagrams revealed that there were 172 differentially expressed LncRNAs in CR vs DN, of which 100 were up-regulated and 72 were down-regulated **(** **Figures 2E–G****)**. There were 40 differentially expressed LncRNAs in DN vs DG, among which 29 were up-regulated and 11 were down-regulated **(** **Figures 2E–G****)**. As shown in **Table 1**, 7 common differentially expressed LncRNAs (NR_015554.2, XR_382492.3, XR_382493.3, XR_382494.3, XR_388840.1, XR_873495.2 and XR_876705.2) were found in the two comparisons, and dapagliflozin could reverse the expression changes of 7 LncRNAs. The top 10 upregulated and downregulated LncRNAs in CR vs DN and DN vs DG respectively was listed in **Tables S2** and **S3**.

**Table 1:** The differentially expressed LncRNAs in the three groups.

Gene ID	Gene symbol	Log2 (fold change)		P-Value	
		CR-DN	DN-DG	CR-DN	DN-DG
NR_015554.2	AI506816	-5.535820015	4.037966831	1.10E-06	0.004185984
XR_382492.3	2010203P06Rik	2.788783699	-1.375859426	0.000886101	0.027335057
XR_382493.3	2010203P06Rik	2.654227803	-1.546562094	0.001974919	0.02068988
XR_382494.3	2010203P06Rik	3.538211533	-1.32722677	0.000306679	0.035530862
XR_873495.2	2010203P06Rik	2.755958903	-1.368824543	0.00120733	0.028809256
XR_388840.1	Gm35001	2.637250895	-2.18855647	0.018245622	0.016443863
XR_876705.2	Gm22146	-1.909626787	1.677984089	0.03972669	0.043126027

CR, Control group; DN, diabetic nephropathy group; DG, Dapagliflozin group.

### Identification of differentially expressed mRNAs in DN

**Figure 3:**
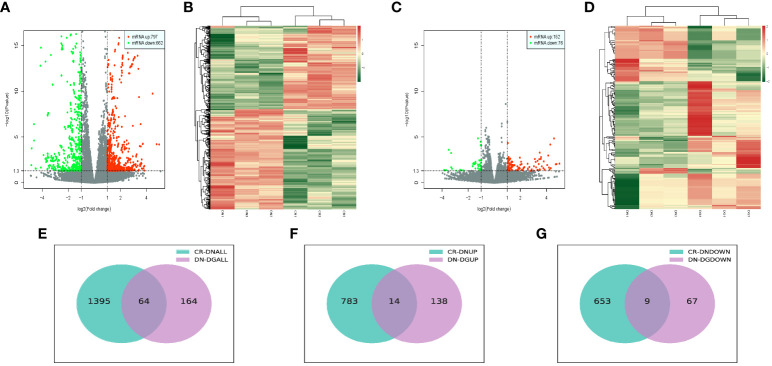
Differentially expressed mRNAs in DN (n=3). **(A, B)** Volcano plots **(A)** and heatmaps **(B)** for comparison between CR and DN. **(C, D)** Volcano plots **(C)** and heatmaps **(D)** for comparison between DN and DG. **(E)** Overlap of the differentially expressed mRNAs in the comparisons between CR vs DN and DN vs DG. **(F)** Upregulated differentially expressed mRNAs in the comparisons between CR vs DN and DN vs DG. **(G)** Downregulated differentially expressed mRNAs in the comparisons between CR vs DN and DN vs DG. CR, control group; DN, diabetic nephropathy group; DG, dapagliflozin group.

We analyzed the differentially expressed mRNAs between groups. Volcano plots and heat map offered an overview of mRNAs differential expressed in CR vs DN and DN vs DG **(** **Figures 3A–D****)**. Compared with CR group, 1459 mRNAs were markedly expressed in the DN group (797 upregulated and 662 downregulated). Compared with DN group, 228 mRNAs were significantly expressed in the DG group (152 upregulated and 76 downregulated) **(** **Figures 3E–G****)**. As shown in Venn diagrams, 64 common differentially expressed mRNAs were found in CR vs DN and DN vs DG. The top 10 upregulated and downregulated mRNAs in CR vs DN and DN vs DG respectively was listed in **Tables S4** and **S5**.

### The co-expression network of LncRNA-mRNA

**Figure 4:**
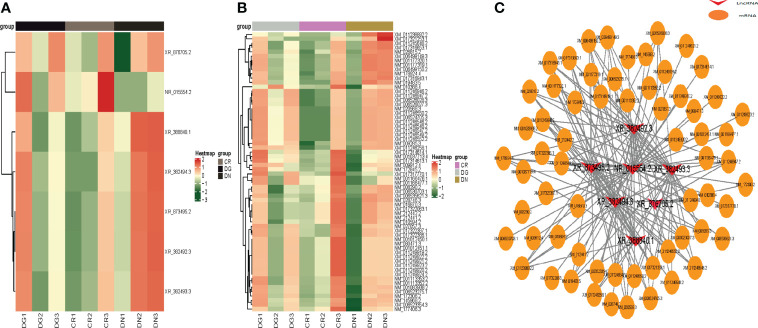
Co-expression network of LncRNA-mRNA. **(A, B)** Heatmap showing clustering analysis of differentially expressed LncRNAs **(A)** and mRNAs **(B)** in CR, DN and DG. **(C)** LncRNA-mRNA co-expression network in 7 LncRNAs and 63 mRNAs. CR, control group; DN, diabetic nephropathy group; DG, dapagliflozin group.

We constructed heatmaps to show the expression patterns of LncRNAs and mRNAs in CR, DN and DG groups **(** **Figures 4A, B****)**. Thus, we build a co-expression network using the common differentially expressed LncRNAs and mRNAs in the three groups. We found that 7 differentially expressed LncRNAs were co-expressed with the 63 differentially expressed mRNAs and may have targeted these genes **(** **Figure 4C****)**.

### Functional enrichment analysis of the differentially expressed mRNAs and LncRNAs

**Figure 5:**
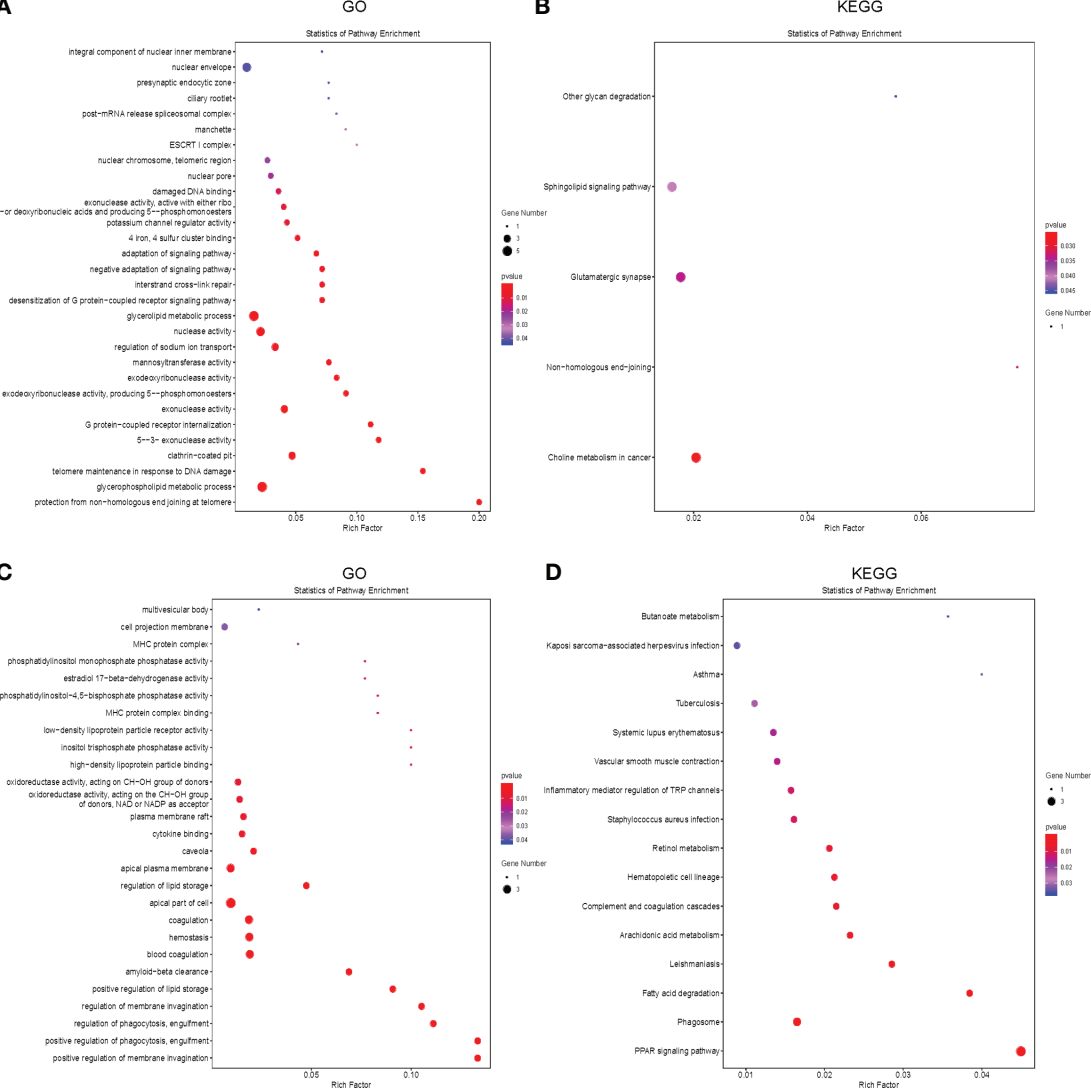
Functional enrichment analysis of the differentially expressed mRNAs and LncRNAs. **(A, B)** The bubble plot showing GO analysis **(A)** and KEGG analysis **(B)** for differentially expressed LncRNAs in CR, DN and DG. **(C, D)** The Bubble plot showing GO analysis **(C)** and KEGG analysis **(D)** for differentially expressed mRNAs in CR, DN and DG, CR, control group; DN, diabetic nephropathy group; DG, dapagliflozin group.

Potential target genes of 7 common differentially expressed LncRNAs (DELncRNAS) in the two comparisons were predicted bioinformatically. GO analysis revealed that these genes were enriched mainly in pathways such as glycerophospholipid metabolic process, telomere maintenance, exonuclease activity, etc **(** **Figure 5A****)**. Then, we annotated these DELncRNA-target mRNA genes by KEGG pathway analysis. The results revealed that the genes were evidently enriched in non−homologous end−joining, glutamatergic synapse, sphingolipid signaling pathway **(** **Figure 5B****)**.

Meanwhile, we performed GO and KEGG analysis to annotate the 63 common differentially expressed mRNAs in the two comparisons. GO analysis revealed that these genes were enriched mainly in positive regulation of membrane invagination, regulation of phagocytosis, positive regulation of lipid storage **(** **Figure 5C****)**. KEGG pathway analysis showed that the genes were evidently enriched in PPAR signaling pathway, phagosome, fatty acid degradation, arachidonic acid metabolism, complement and coagulation cascades, etc **(** **Figure 5D****)**.

### Identification of candidate targets of dapagliflozin in the treatment of DN by network pharmacology

**Figure 6:**
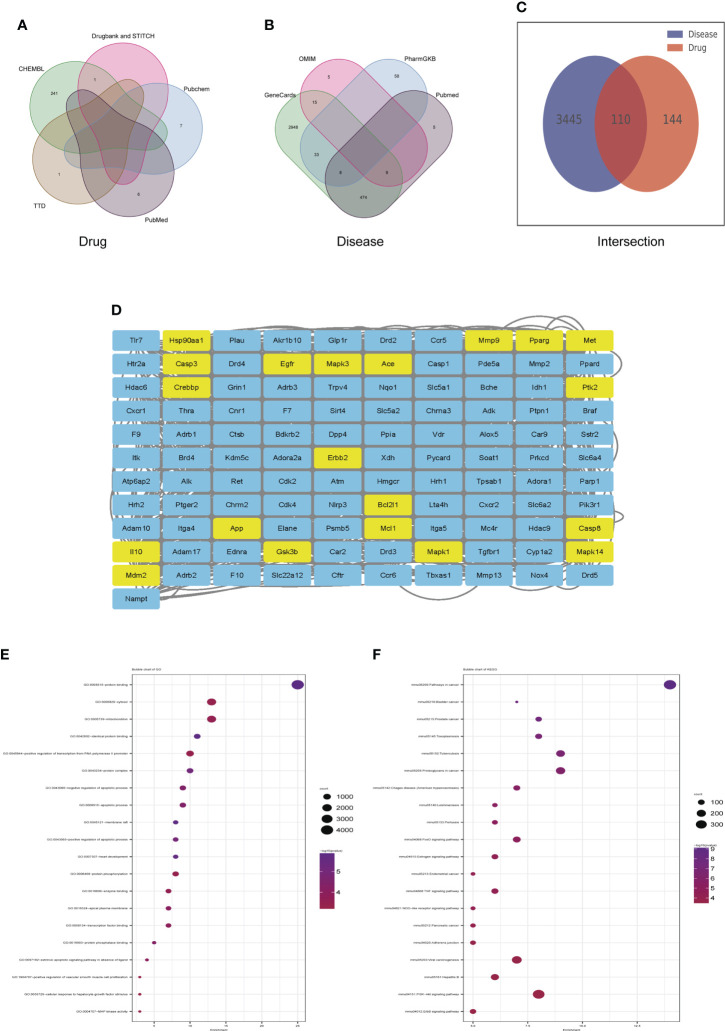
Identification of candidate targets of Dapagliflozin in the treatment of DN by network pharmacology. **(A)** The Veen diagram showing candidate targets of dapagliflozin from the drug database. **(B)** The Veen diagram showing candidate targets of DN from disease databases. **(C)** Overlap of the differentially expressed genes between candidate targets of dapagliflozin and DN. **(D)** PPI network of the 110 overlapping genes between candidate targets of dapagliflozin and DN. The top 20 core genes were highlighted in yellow. **(E, F)** GO **(E)** and KEGG **(F)** enrichment analysis of the top 20 core genes. DN, diabetic nephropathy.

Veen diagram showed that 255 genes candidate targets of dapagliflozin were searched out from the drug database **(** **Figure 6A****)** and 3555 candidate genes of DN were collected from disease databases **(** **Figure 6B****)**. After intersection, a total of 110 overlapping genes were identified **(** **Figure 6C****)**. To estimate the role of the therapeutic target genes, the 110 overlapping genes were used to construct PPI network and were sorted in descending order by degree with the topology parameters of PPI network **(** **Figure 6D****)**. The top 20 core genes were submitted to perform GO and KEGG enrichment analysis, which revealed that these genes were enriched mainly in MAPK signaling, PPAR signaling pathway and PI3k-Akt signaling pathway, etc **(** **Figures 6E, F****)**.

### Component- target-pathway network construction

**Figure 7:**
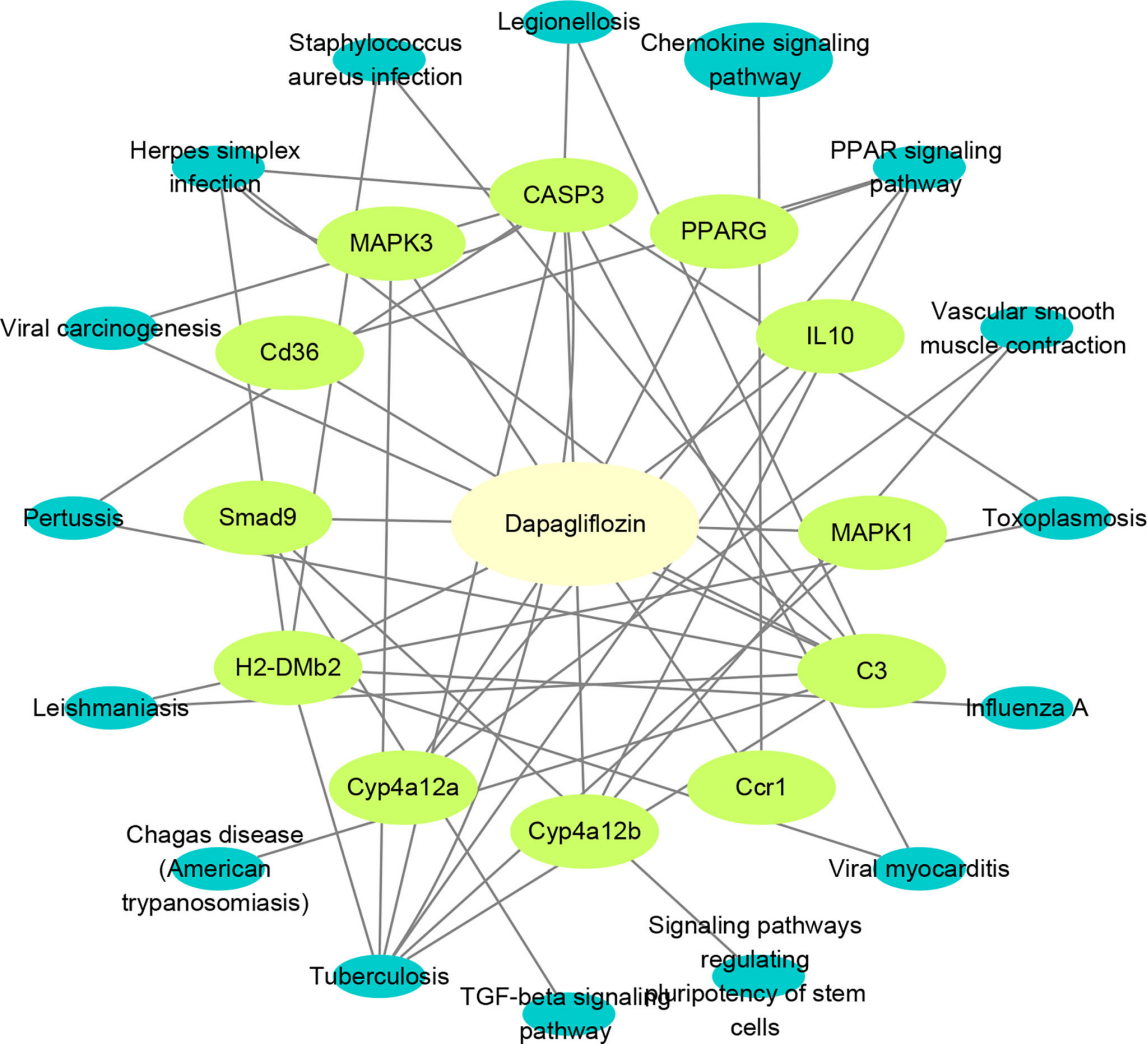
Construction of component-target-pathway network. Light yellow represents dapagliflozin, light green represents potential targets and dark blue represents gene-related signaling pathways.

To more accurately identify the mechanisms of dapagliflozin for DN, we performed KEGG enrichment analysis using the 63 mRNAs involved in LncRNA-mRNA co-expression network and the 20 core genes identified by network pharmacology. Pathways that the above two sets of genes are commonly involved in are considered to play important roles. The commonly involved pathways are including TGF-beta signaling pathway, Tuberculosis, Chagas disease (American trypanosomiasis), Leishmaniasis, Pertussis, Viral carcinogenesis, Herpes simplex infection, Staphylococcus aureus infection, Legionellosis, Chemokine signaling pathway, PPAR signaling pathway, Vascular smooth muscle contraction, Toxoplasmosis, Influenza A,Viral myocarditis and Signaling pathways regulating pluripotency of stem cells. Then, we used Cytoscape3.8.2 to construct component-target-pathway network based on the data of KEGG enrichment analysis of RNA Sequencing and Network Pharmacology **(** **Figure 7****)**. We found that CD36, SMAD9, H2-DMB2, CYP4A12a, CYP4A12b, Ccr1, C3, MAPK1, MAPK3, CASP3, PPARG, IL10 might be the key targets for dapagliflozin in treating DN and these genes were evidently enriched in pathways including PPAR signaling pathway, Chemokine signaling pathway, TGF-β signaling pathway, etc.

### Validation of key LncRNAs and genes by qRT-PCR and western blotting

**Figure 8:**
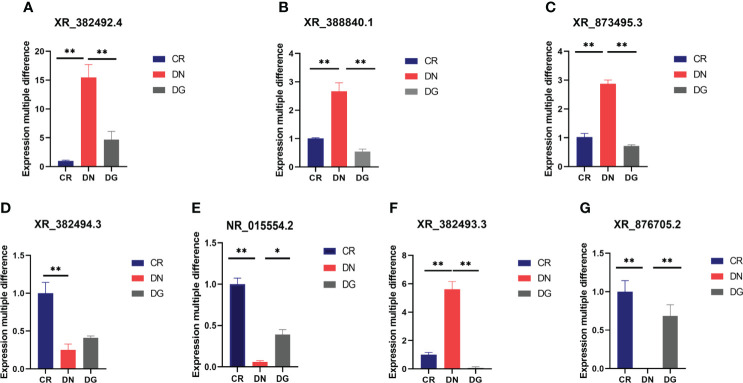
**(A–G)** Validation of key LncRNAs by qRT-PCR (n=6). Data are presented as the mean ± SE. *P < 0.05; **P < 0.001. CR, control group; DN, diabetic nephropathy group; DG, dapagliflozin group.

To verify the RNA sequencing and network pharmacology results, seven LncRNAs and twelve mRNAs were performed by qRT-PCR in the kidneys from the three groups. We found that XR_382492.4, XR_873495.3, XR-388840.1 and XR_382493.3 were markedly upregulated and NR-015554.2, XR_382494.3 and XR-876705.2 were significantly downregulated in DN group compared with CR group. Dapagliflozin can reverse the expression changes of XR_382492.4, XR_873495.3, XR-388840.1, NR-015554.2, XR-382493.3 and XR-876705.2 **(** **Figure 8****)**. Dapagliflozin tended to increase the expression of XR_382494.3, but this effect failed to reach statistical significance.

**Figure 9:**
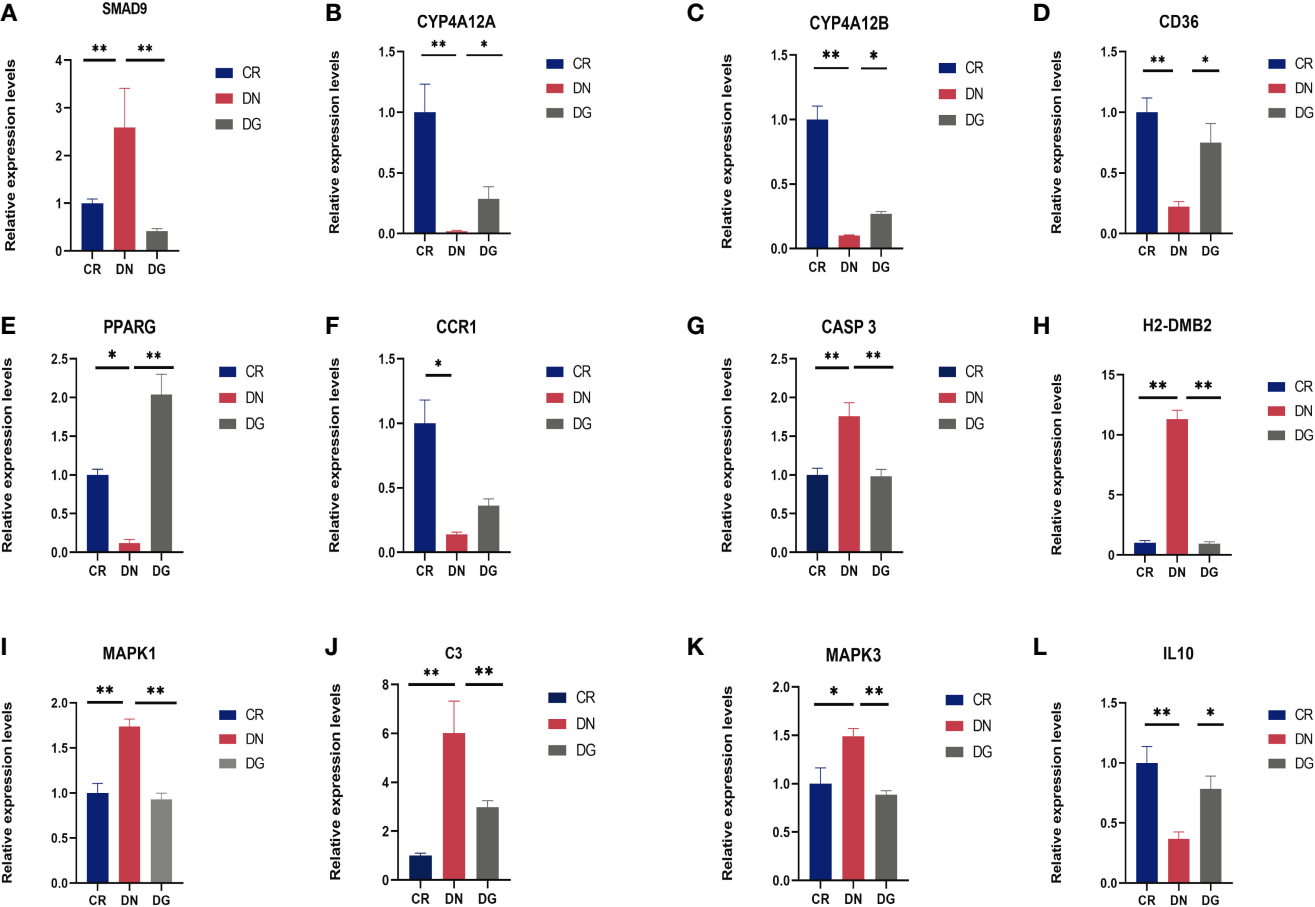
**(A-L)** Validation of key targets by qRT-PCR (n=6). Data are presented as the mean ± SE. *P < 0.05; **P < 0.001. CR, control group; DN, diabetic nephropathy group; DG, dapagliflozin group.

**Figure 10:**
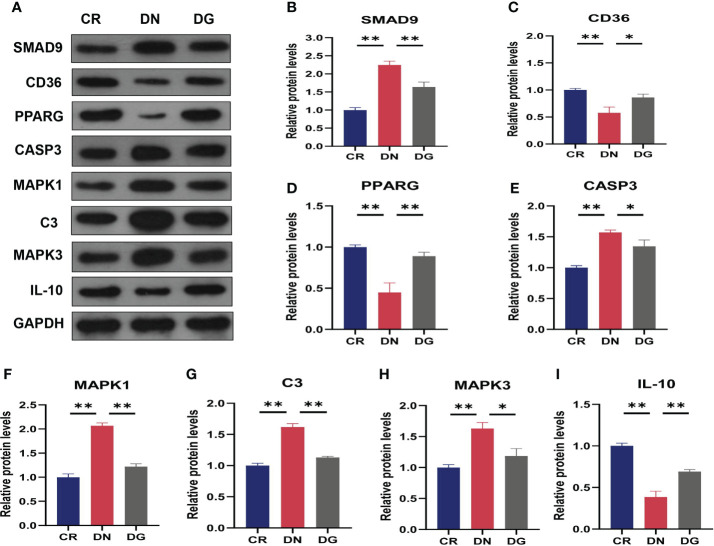
Validation of key targets by western blotting (n=3). **(A)** The protein expression of key targets was detected by western blotting. **(B–I)** Densitometry analysis of western blotting. Data are presented as the mean ± SE. *P < 0.05; **P < 0.001. CR, control group; DN, diabetic nephropathy group; DG, dapagliflozin group.

Meanwhile, mRNA expression of SMAD9, CASP3, H2-DMB2, MAPK1, MAPK3 and C3 were upregulated and CYP4A12A, CYP4A12B, CD36, PPARG, and IL-10 were downregulated in DN group compared with CR group. Dapagliflozin can reverse the expression changes of all these genes **(** **Figure 9****)**. CCR1 was decreased in DN group compared with CR group and dapagliflozin tended to increase the expression of CCR1 but it failed to achieve the statistical level. As shown in **Figure 10**, protein expression of SMAD9, CASP3, MAPK1, MAPK3 and C3 were markedly increased and PPARG, CD36, and IL-10 were significantly decreased in DN group compared with CR group. Dapagliflozin can reverse the expression changes of all these proteins. The results indicated that SMAD9, CASP3, H2-DMB2, MAPK1, MAPK3, C3, CYP4A12A, CYP4A12B, CD36, PPARG, and IL-10 maybe potential targets of Dapagliflozin in DN.

## Discussion

DN is the main cause of ESKD worldwide (19). Identifying novel molecular mechanisms and targets underlying DN progression will be beneficial for developing novel therapeutic approaches (20). Dapagliflozin has been shown to have protective effect in cardiomyopathy and kidney disease, especially against complications associated with diabetes (21, 22). Clinical evidence has confirmed that the renoprotective effect of dapagliflozin goes beyond glucosuric effect, which are not fully elucidated. In addition, dapagliflozin can not only improve glycemic control, but also reduce body weight and lower blood pressure. We proposed that dapagliflozin may protect against DN through other unknown effects. Previous studies suggested that dapagliflozin improved albuminuria and tubulointerstitial fibrosis in DN *via* suppressing SGK1 and reversing the T-cell imbalance (23). A new report indicated that dapagliflozin exerts protective effects on DN by reducing cellular senescence and inhibiting oxidative stress (24). However, these studies mainly focused on specific molecular signaling pathways, no studies explored the whole transcriptome changes of dapagliflozin in DN mice. Here, we used RNA sequencing and network pharmacology to systematically disclose the mechanisms of dapagliflozin in the treatment of DN.

Emerging studies have investigated the role of LncRNAs in DN (25–27). However, this is the first study exploring LncRNAs of dapagliflozin in treating DN. We identified 172 differentially expressed LncRNAs in CR vs DN and 40 differentially expressed LncRNAs in DN vs DG. 7 common differentially expressed LncRNAs (NR_015554.2, XR_382492.3, XR_382493.3, XR_382494.3, XR_388840.1, XR_873495.2 and XR_876705.2) were found in the two comparisons, and results of qRT-PCR revealed that dapagliflozin can reverse the expression changes of XR_382492.4, XR_873495.3, XR-388840.1, NR-015554.2, XR-382493.3 and XR-876705.2. Of these novel LncRNAs, only NR_015554.2 (LncRNA AI506816) has been reported, which was related with multiparity (28), whereas the current state of evidence for other LncRNAs has so far been unknown. Meanwhile, our data revealed that 63 mRNAs were common differentially expressed in CR vs DN and DN vs DG. Then, we constructed a lncRNA-mRNA co-expression network, which is a powerful tool to predict the function of LncRNA. The co-expression network identified 7 lncRNAs and 63 mRNAs involving in the core co-expression network. These genes were related to PPAR signaling pathway, phagosome, fatty acid degradation, arachidonic acid metabolism, complement and coagulation cascades. A recent study showed that dapagliflozin can enhanced fatty acid metabolism in DN mice (29). Dapagliflozin was demonstrated to ameliorate DN by promoting Crry and inhibiting complement over-activation in diabetic model (30). These reports supported our results.

Furthermore, in order to find the hub genes involved in the protective effect of dapagliflozin in the treatment of DN, we used network pharmacology for predicting the therapeutic targets of dapagliflozin and performed integrated analysis based on the data of RNA sequencing and network pharmacology. We found 110 differentially expressed overlapping genes between candidate targets of dapagliflozin and DN and the top 20 core genes were obtained by PPI network. We further construct component-target-pathway network and collected 11 hub genes including SMAD9, CYP4A12a, CYP4A12b, CD36, PPARG, CASP3, H2-DMB2, MAPK1, C3, MAPK3, IL10, which were mainly enriched in TGF-β signaling pathway, PPAR signaling pathway and chemokine signaling pathway. TGF-β family signaling plays a vital role in the regulation of cell growth, differentiation, and development, especially in fibrosis in many organ systems (31). SMADs are key intracellular transducers which transduces signals from TGF-β family members. SMAD9, also known as SMAD8, was reported to be involved in diabetic renal tubulointerstitial fibrosis (32). A recent study revealed that SMAD9 was highly expressed in the blood of diabetes patients and in streptozotocin-induced rat retinas, which indicated that SMAD9 was closely related with diabetic retinopathy (33). SMAD9 was also reported to be expressed in human kidneys (34). Similarly, our results revealed that SMAD9 was increased in DN mice and dapagliflozin can downregulate the expression of SMAD9, indicating that SMAD9 may be a potential target of dapagliflozin for DN. Peroxisome proliferators-activated receptors (PPARs), a group of nuclear proteins that including PPAR-alpha, PPAR-beta/delta and PPAR-gamma, have been implicated in the regulation of gene transcription and metabolic processes (35). Studies have demonstrated that members of the cytochrome P-450 4 (CYP4) family have the PPAR response element and can be regulated by PPAR-alpha (36). CYP4 proteins can convert arachidonic acid (AA) to 20-hydroxyeicosatetraenoic acids (20-HETE), which can either reduce albuminuria or cause injury by promoting podocyte apoptosis or tubular hypertrophy (37, 38). In the present study, CYP4A12a and CYP4A12b were found to be decreased in the DN mice and dapagliflozin can increase the expression of the two genes. We speculated that the downregulation of CYP4A12a and CYP4A12b cause reduced synthesis of 20-HETE, leading to impaired kidney function. A previous study indicated that the mRNA levels of CYP4A12a and CYP4A12b are decreased in renal cortex tissues from db/db mice, which is consistent with our results (39). PPAR-gamma (PPARG) plays a critical role in adipogenesis and insulin sensitivity and PPARG polymorphism contributes to the development of DN in diabetic patients (40). Thiazolidinedione (TZD), a high-affinity synthetic ligand for PPARG, has been demonstrated to improve insulin resistance, reduce proteinuria and ameliorate renal function in diabetic nephropathy (41). In addition, studies reported that Pparg null-mice present increased glucosuria, albuminuria, decreased creatinine clearance and mesangial expansion (42).Our data also showed that PPARG was significantly downregulated in the DN mice and dapagliflozin can upregulate the expression of PPARG. Scavenger receptor CD36, also known as fatty acid translocase (FAT), is a surface glycoprotein. It can function in many processes including fatty acid metabolism, apoptosis, angiogenesis, phagocytosis and inflammation and act as a transcriptional regulator of PPARG (43). It has been reported that CD36 can be a plausible prognostic marker for DN (44). Currently, the regulatory effect of CD36 on insulin resistance is still controversial. Studies suggested that overexpression of CD36 can promotes the development of metabolic syndrome and insulin resistance (45), while CD36-deficient patients presented impaired glucose metabolism, insulin resistance and hyperlipidemia (46). Loss of CD36 deficiency can lead to hepatic insulin resistance and impair hepatic insulin signaling in mice fed a low-fat diet (47). A recent study showed that CD36 was highly expressed in renal tubules in mice fed a high-fat diet (48). However, according to our findings, db/db mice presented downregulated expression level of CD36 and dapagliflozin can reverse the expression changes. We speculated the expression levels of CD36 may be affected by different nutritional status, which partly explains CD36 plays a contradictory role in glucose metabolism. The above results indicated that dapagliflozin exerts protective effects on DN through PPAR signaling pathway involving PPARG, CYP4A12a, CYP4A12b and CD36, which have not been previously reported. Significantly, the genes identified by the lncRNA-mRNA co-expression network were also enriched in PPAR signaling pathway, which regulates metabolic homeostasis, lipid, glucose and energy metabolism. Thiazolidinedione, act as PPARG agonist, was limited used in patients with diabetes due to its effect of increasing sodium reabsorption, leading to fluid retention and edema (49). Thus, considering the effect of sodium excreting and activating PPARG, patients with DN may benefit more from combination of dapagliflozin and thiazolidinedione than a single therapy, which was supported by previous studies (50).

In addition, we found that MAPK1, MAPK3, C3, CASP3 and H2-DMB2 were increased in db/db mice, whereas IL-10 were decreased in db/db mice and dapagliflozin can reverse the expression of these genes. Previous studies concluded that dapagliflozin protected against DN and reduced urinary albumin excretion *via* reducing the expression of MAPK signaling pathways (51, 52). Immune inflammation plays a pivotal role in the pathogenesis of DN. Complement C3 was proved to be negatively related to the glomerular filtration rate in patients with DN and could be immune-related biomarkers of DN (53). It has been reported that dapagliflozin can attenuate complement over-activation and upregulate the anti-inflammatory cytokine IL-10 in diabetic mice (30, 54), which consisted with our results again. Recent studies showed that CASP3 was upregulated in diabetic rats and diabetic human kidney tubuli (55, 56). However, there are lack of evidence about relationships between CASP3 and dapagliflozin. H2-DMB2, playing a key role in antigen presentation by MHC class II molecules, has not been studied in DN.

Notably, in order to verify whether these hub genes play the same role in human, we searched human gene-disease databases including DisGeNET, GeneCardSuite and OMIM to screen the genes related with DN. We found that PPARG, CD36, MAPK1, MAPK3, CASP3, C3 and IL10 were related with DN, while the evidence of SMAD9, CYP4A12a, CYP4A12b and H2-DMB2 in DN was absent. Therefore, these results may provide us with new possible mechanisms mediating the pathogenesis of DN and also offer us new potential targets of dapagliflozin for DN.

Although we systematically elucidated the underlying molecular mechanisms of dapagliflozin in the treatment of DN, there were still several limitations in our study. First, the sample size in each group submitted for RNA sequencing was limited. Second, detailed interactions of the co-expressed lncRNAs and mRNAs are needed in future studies. Third, the functions of the hub genes obtained by RNA sequencing and network pharmacology need to be further verified *in vivo* and *in vitro*.

In conclusion, we combined RNA sequencing and network pharmacology to explore the potential mechanisms of dapagliflozin in DN. Our study demonstrated that dapagliflozin might treat DN through regulating TGF-β signaling pathway, PPAR signaling pathway and chemokine signaling pathway by targeting 11 hub genes (SMAD9, PPARG, CD36, CYP4A12A, CYP4A12B,CASP3, H2-DMB2, MAPK1, MAPK3, C3, IL-10). Our research provides new insights into the protective mechanism of dapagliflozin for DN.

## Data availability statement

The data presented in the study are deposited in the Sequence Read Archive (SRA) repository, accession numbers SRR19792050, SRR19792049, SRR19792048, SRR19792047, SRR19792046, SRR19792045, SRR19792044, SRR19792043 and SRR19792042.

## Ethics statement

The animal study was reviewed and approved by The Animal Experiment Ethics Committee of Jinan University.

## Author contributions

YX, XZ, and LC conceived and designed the study; ZB, TX, and TL conducted the experiments and obtained the data; ZC, LY, CZ, and JL analyzed and collated the data; YX and ZB drafted and written the final version of the manuscript. ZB, TX, and TL contributed equally to this work. All authors approved the final version of the manuscript.

## Funding

This work was supported by the National Natural Science Foundation of China (no.82074307, no.82174148), the Natural Science Foundation of Guangdong Province, China (no.2018030310451) and Wuxi Municipal Health Commission Scientific Research Fund Youth Project (Q202106).

## Conflict of interest

The authors declare that the research was conducted in the absence of any commercial or financial relationships that could be construed as a potential conflict of interest.

## Publisher’s note

All claims expressed in this article are solely those of the authors and do not necessarily represent those of their affiliated organizations, or those of the publisher, the editors and the reviewers. Any product that may be evaluated in this article, or claim that may be made by its manufacturer, is not guaranteed or endorsed by the publisher.
