# Supplementary material for: An integrated RNA sequencing and network pharmacology approach reveals the molecular mechanism of dapagliflozin in the treatment of diabetic nephropathy
Source: Front Endocrinol (Lausanne). 2022 Sep 21;13:967822. doi: 10.3389/fendo.2022.967822 (PMC9533015; doi:10.3389/fendo.2022.967822)
Supplement: Supplementary file 1 [file Table_1.docx]

**Table S1**. Primers of 7 LncRNAs and 12 genes used for qRT-PCR

| \| **Primer** \| **Forward** \| **Revrse** \| \| --- \| --- \| --- \| \| NR_015554.2 \| TGATGAGTGCTGTGTCCAGG \| TGGAAGTCGGACCAGTTCAC \| \| XR_382492.3 \| GAGCCCTTGACTGGTGATTCT \| CCCTTTCCCACTCCTGTATCTT \| \| XR_382493.3 \| TAGCCAGCCTTGTCTGATACCA \| TGAGTTTGAAGAGACTACCTGGATG \| \| XR_382494.3 \| AGTCACAGACCAAGCCTCTGC \| GCATAGCCCAAGATGTCTCCTC \| \| XR_388840.1 \| GCCATCAGGTCCCAGTCACA \| TGGAGCAGAACTTCGGGTG \| \| XR_873495.2 \| GTGAGAACTGGAGCTTGACCC \| GATGTCCTGGGTTGAATATGGAC \| \| XR_876705.2 \| AGTCACAGACCAAGCCTCTGC \| CCCAGGTGATCATAGAATTATCATCC \| \| C3 \| CCAGCTCCCCATTAGCTCTG \| GCACTTGCCTCTTTAGGAAGTC \| \| CASP3 \| CTGACTGGAAAGCCGAAACTC \| CGACCCGTCCTTTGAATTTCT \| \| Ccr1 \| CTCATGCAGCATAGGAGGCTT \| ACATGGCATCACCAAAAATCCA \| \| CD36 \| ATGGGCTGTGATCGGAACTG \| GTCTTCCCAATAAGCATGTCTCC \| \| Cyp4a12a \| ATGGGCTGTGATCGGAACTG \| GTCTTCCCAATAAGCATGTCTCC \| \| Cyp4a12b \| CTGGATCTTCTATGCTT \| GCTCTCTGCTCACACTT \| \| H2-DMB2 \| GGATGAAGAATGGGCAG \| CCGTAGGAAGGGGTTAG \| \| IL10 \| CTTACTGACTGGCATGAGGATCA \| GCAGCTCTAGGAGCATGTGG \| \| MAPK1 \| CAGGTGTTCGACGTAGGGC \| TCTGGTGCTCAAAAGGACTGA \| \| MAPK3 \| ATCTGCTTATCAACACCACCTG \| CGGTTGGAGAGCATCTCAGC \| \| PPARG \| GGAAGACCACTCGCATTCCTT \| GTAATCAGCAACCATTGGGTCA \| \| Smad9 \| CGGGTCAGCCTAGCAAGTG \| GAGCCGAACGGGAACTCAC \| |
| --- | --- | --- | --- | --- | --- | --- | --- | --- | --- | --- | --- | --- | --- | --- | --- | --- | --- | --- | --- | --- | --- | --- | --- | --- | --- | --- | --- | --- | --- | --- | --- | --- | --- | --- | --- | --- | --- | --- | --- | --- | --- | --- | --- | --- | --- | --- | --- | --- | --- | --- | --- | --- | --- | --- | --- | --- | --- | --- | --- | --- |
|  |
|  |
