# Supplementary material for: An integrated RNA sequencing and network pharmacology approach reveals the molecular mechanism of dapagliflozin in the treatment of diabetic nephropathy
Source: Front Endocrinol (Lausanne). 2022 Sep 21;13:967822. doi: 10.3389/fendo.2022.967822 (PMC9533015; doi:10.3389/fendo.2022.967822)
Supplement: Supplementary file 2 [file Table_2.docx]

**Table S2.**TOP 10 upregulated and downregulated LncRNAs in CR vs DN

| **Gene ID** | **Gene symbol** | **log2(Fold_change)** | **p-value** | **Style** |  |
| --- | --- | --- | --- | --- | --- |
| ENSMUST00000194648.1 | ENSMUSG0000103706.1 | 7.572744514 | 3.09201E-16 | UP | |
| NR_033305.1 | AA388235 | 6.220935405 | 1.63892E-07 | UP | |
| XR_872234.2 | Gm40812 | 6.045934037 | 0.002674732 | UP | |
| XR_378102.1 | Gm29930 | 5.703683085 | 3.28003E-07 | UP | |
| XR_872234.2 | Gm40812 | 5.407829239 | 0.004659168 | UP | |
| XR_872749.1 | Gm35071 | 5.086089035 | 0.000114351 | UP | |
| XR_872750.1 | Gm35071 | 5.012228093 | 0.000163101 | UP | |
| ENSMUST00000193906.1 | ENSMUSG00000103951.1 | 4.961033181 | 5.00278E-05 | UP | |
| XR_381586.2 | Gm35071 | 4.811032143 | 0.000463487 | UP | |
| XR_876866.1 | Gm41595 | 4.651520315 | 0.000166323 | UP | |
| XR_872578.2 | Gm36129 | -1.003642131 | 0.00159884 | DOWN | |
| XR_872575.2 | Gm36129 | -1.003960349 | 0.001782834 | DOWN | |
| ENSMUST00000198500.1 | ENSMUSG00000106535.1 | -1.011623832 | 0.027907243 | DOWN | |
| ENSMUST00000213859.1 | ENSMUSG00000110755.1 | -1.04452016 | 0.016598377 | DOWN | |
| ENSMUST00000147504.1 | ENSMUSG00000055370.3 | -1.059804633 | 2.82672E-05 | DOWN | |
| ENSMUST00000217474.1 | ENSMUSG00000110755.1 | -1.064085136 | 0.011816939 | DOWN | |
| XR_001782015.1 | LOC108167374 | -1.083715384 | 0.029469586 | DOWN | |
| ENSMUST00000181957.1 | ENSMUSG00000097312.1 | -1.094603507 | 0.010204299 | DOWN | |
| NR_126558.1 | E230013L22Rik | -1.102617282 | 0.049294867 | DOWN | |
| NR_015503.1 | A330040F15Rik | -1.115485784 | 0.028590168 | DOWN | |

CR: Control group; DN: diabetic nephropathy group; DG: Dapagliflozin group
