# Supplementary material for: An integrated RNA sequencing and network pharmacology approach reveals the molecular mechanism of dapagliflozin in the treatment of diabetic nephropathy
Source: Front Endocrinol (Lausanne). 2022 Sep 21;13:967822. doi: 10.3389/fendo.2022.967822 (PMC9533015; doi:10.3389/fendo.2022.967822)
Supplement: Supplementary file 3 [file Table_3.docx]

**Table S3.**TOP 10 upregulated and downregulated LncRNAs in DN vs DG

| **Gene ID** | **Gene symbol** | **log2(Fold_change)** | **p-value** | **Style** |  |
| --- | --- | --- | --- | --- | --- |
| XR_001779143.1 | LOC102631930 | 1.029727686 | 0.000783101 | UP | |
| XR_001780601.1 | Gm34408 | 1.101717855 | 0.013706987 | UP | |
| NR_131212.1 | Neat1 | 1.1057864 | 9.4846E-17 | UP | |
| XR_001780601.1 | Gm34408 | 1.148224626 | 0.009509616 | UP | |
| XR_877637.1 | Gm31909 | 1.201545436 | 0.02598709 | UP | |
| XR_879222.1 | Gm31278 | 1.205608092 | 0.048632034 | UP | |
| XR_390555.3 | Gm32657 | 1.249286931 | 0.015554949 | UP | |
| XR_001779765.1 | Gm38485 | 1.252576515 | 0.009309828 | UP | |
| XR_001780601.1 | Gm34408 | 1.263580653 | 0.005459317 | UP | |
| ENSMUST00000200128.1 | ENSMUSG00000104936.1 | 1.374061548 | 0.039102778 | UP | |
| XR_001778646.1 | OC108167550 | -3.873101288 | 0.035337196 | DOWN | |
| XR_880343.1 | Gm39482 | -3.656329976 | 0.024083095 | DOWN | |
| ENSMUST00000135670.1 | ENSMUSG00000055134.5 | -2.714415417 | 0.015208536 | DOWN | |
| ENSMUST00000196922.1 | ENSMUSG00000104748.1 | -2.633983098 | 0.027863949 | DOWN | |
| XR_388840.1 | Gm35001 | -2.18855647 | 0.016443863 | DOWN | |
| XR_870958.2 | Gm27216 | -1.820345034 | 0.017235157 | DOWN | |
| XR_382493.3 | 2010203P06Rik | -1.546562094 | 0.02068988 | DOWN | |
| XR_879464.1 | Gm42060 | -1.476098088 | 0.02409436 | DOWN | |
| XR_382492.3 | 2010203P06Rik | -1.375859426 | 0.027335057 | DOWN | |
| XR_873495.2 | 2010203P06Rik | -1.368824543 | 0.028809256 | DOWN | |

CR: Control group; DN: diabetic nephropathy group; DG: Dapagliflozin group
