# Supplementary material for: An integrated RNA sequencing and network pharmacology approach reveals the molecular mechanism of dapagliflozin in the treatment of diabetic nephropathy
Source: Front Endocrinol (Lausanne). 2022 Sep 21;13:967822. doi: 10.3389/fendo.2022.967822 (PMC9533015; doi:10.3389/fendo.2022.967822)
Supplement: Supplementary file 4 [file Table_4.docx]

**Table S4.** TOP 10 upregulated and downregulated mRNAs in CR vs DN

| **Gene ID** | **Gene symbol** | **log2(Fold_change)** | **p-value** | **Style** |  |
| --- | --- | --- | --- | --- | --- |
| NM_199477.2 | Mettl7a2 | 8.476731009 | 7.6432E-37 | UP | |
| NM_013697.5 | Ttr | 7.312217892 | 0.000104187 | UP | |
| XM_011245712.2 | Mettl7a3 | 6.513324229 | 7.48724E-09 | UP | |
| XM_006535833.3 | Gm29779 | 5.67277553 | 0.000153272 | UP | |
| XM_011238892.2 | Gm39701 | 4.974774182 | 6.45146E-05 | UP | |
| NM_001002900.1 | Higd1c | 4.780885967 | 6.05804E-05 | UP | |
| NM_001024672.3 | Methig1 | 4.474631997 | 1.75542E-10 | UP | |
| XM_006510814.2 | Gsta2 | 4.20170098 | 4.83907E-36 | UP | |
| NM_008182.3 | Gsta2 | 4.166636564 | 3.7104E-36 | UP | |
| NM_001166544.1 | Hmga1 | 3.988254668 | 1.34829E-18 | UP | |
| XM_017314030.1 | Chst3 | -1.00142994 | 0.025290252 | DOWN | |
| XM_006512114.1 | Acaa1b | -1.00257747 | 1.57239E-09 | DOWN | |
| XM_006538310.3 | Stra6l | -1.004463491 | 0.016594632 | DOWN | |
| XM_006538311.3 | Stra6l | -1.004463491 | 0.016594632 | DOWN | |
| XM_011243512.2 | Chst3 | -1.008991538 | 0.025656452 | DOWN | |
| NM_010023.4 | Eci1 | -1.011317597 | 8.53279E-11 | DOWN | |
| NM_145987.2 | Tmem82 | -1.011740394 | 0.004570351 | DOWN | |
| XM_017322429.1 | 1700016C15Rik | -1.012319245 | 0.002592906 | DOWN | |
| NM_001195596.1 | Smlr1 | -1.013027668 | 0.034234131 | DOWN | |
| NM_028788.4 | Stra6l | -1.013230567 | 0.015985094 | DOWN | |

CR: Control group; DN: diabetic nephropathy group; DG: Dapagliflozin group
