# Supplementary material for: An integrated RNA sequencing and network pharmacology approach reveals the molecular mechanism of dapagliflozin in the treatment of diabetic nephropathy
Source: Front Endocrinol (Lausanne). 2022 Sep 21;13:967822. doi: 10.3389/fendo.2022.967822 (PMC9533015; doi:10.3389/fendo.2022.967822)
Supplement: Supplementary file 5 [file Table_5.docx]

**Table S5**. TOP 10 upregulated and downregulated mRNAs in DN vs DG

| **Gene ID** | **Gene symbol** | **log2(Fold_change)** | **p-value** | **Style** |  |
| --- | --- | --- | --- | --- | --- |
| NM_011260.1 | Reg3g | 6.297147197 | 0.023215028 | UP | |
| NM_009140.2 | Cxcl2 | 5.889427601 | 0.004767307 | UP | |
| NM_146017.3 | Gabrp | 5.674554385 | 0.013550146 | UP | |
| XM_006514666.3 | Gabrp | 5.581108481 | 0.042019351 | UP | |
| NM_001024230.2 | Gm5431 | 5.0010682 | 0.008047687 | UP | |
| NM_001039701.3 | Il1rn | 4.775759575 | 0.009039242 | UP | |
| NM_001159562.1 | Il1rn | 4.716252961 | 0.009414572 | UP | |
| NM_031167.5 | Il1rn | 4.716252961 | 0.009414572 | UP | |
| XM_006497727.2 | Il1rn | 4.716252961 | 0.009414572 | UP | |
| NM_001281852.1 | S100a9 | 4.585438241 | 1.40811E-05 | UP | |
| XM_006499149.3 | Tfpi | -1.003762465 | 0.006335339 | DOWN | |
| XM_006534564.3 | P2rx5 | -1.012568513 | 0.024871838 | DOWN | |
| XM_017322063.1 | Acsm3 | -1.013101271 | 0.003428022 | DOWN | |
| NM_212441.2 | Acsm3 | -1.013925587 | 0.003269607 | DOWN | |
| NM_212442.2 | Acsm3 | -1.014396578 | 0.003461621 | DOWN | |
| NM_010023.4 | Acsm3 | -1.01857697 | 0.003338214 | DOWN | |
| XM_006509754.3 | 1-Mar | -1.029095541 | 0.029200484 | DOWN | |
| XM_006531205.2 | Scoc | -1.042158526 | 7.93125E-05 | DOWN | |
| NM_001285992.1 | Scoc | -1.043747532 | 3.22958E-05 | DOWN | |
| NM_010594.2 | Kap | -1.046774256 | 0.041048426 | DOWN | |

CR: Control group; DN: diabetic nephropathy group; DG: Dapagliflozin group
